# Fine Localization of Acetylcholinesterase in the Synaptic Cleft of the Vertebrate Neuromuscular Junction

**DOI:** 10.3389/fnmol.2018.00123

**Published:** 2018-04-19

**Authors:** Edna Blotnick-Rubin, Lili Anglister

**Affiliations:** Department of Medical Neurobiology, Institute for Medical Research—Israel-Canada (IMRIC), Hebrew University-Hadassah Medical School, Jerusalem, Israel

**Keywords:** nanogold, acetylcholinesterase, basal lamina, synaptic cleft, postjunctional folds

## Abstract

Acetylcholinesterase (AChE) is concentrated at cholinergic synapses, where it is a major factor in controlling the duration of transmitter action. The concentration and localization of AChE within the synaptic cleft are in keeping with the functional requirements of the particular type of synapse. The densities of synaptic AChE at various neuromuscular junctions (NMJs) had been evaluated by quantitative EM-autoradiography using radiolabeled probes. Yet, fundamental issues concerning the precise distribution and location of the enzyme in the cleft remained open: whether and to what extent synaptic AChE is associated with pre- or postsynaptic membranes, or with synaptic basal lamina (BL), and whether it occurs only in the primary cleft (PC) or also in postjunctional folds (PJFs). Nanogold-conjugates of fasciculin, an anticholinesterase polypeptide toxin, were prepared and used to label AChE at NMJs of mouse and frog muscles. Selective intense labeling was obtained at the NMJs, with gold-labeled AChE sites distributed over the BL in the PC and the PJFs. Quantitative analysis demonstrated that AChE sites are almost exclusively located on the BL rather than on pre- or postsynaptic membranes and are distributed in the PC and down the PJFs, with a defined pattern. This localization pattern of AChE is suggested to ensure full hydrolysis of acetylcholine (ACh) bouncing off receptors, thus eliminating its unnecessary detrimental reattachment.

## Introduction

Acetylcholinesterase (AChE) is concentrated at neuromuscular junctions (NMJs) and other cholinergic synapses in the central and peripheral nervous systems. Its required functional role at a given type of synapse is determined by its concentration and distribution within the synaptic cleft. Traditionally, the density of AChE at NMJs was evaluated by quantitative EM-autoradiography using a ^3^H-labeled covalent organophosphate inhibitor (Salpeter, 1967, 1969; Rogers and Salpeter, 1969; Salpeter et al., 1972, 1978; Anglister et al., 1994b). Subsequently, we developed an ^125^I-labeled probe that utilizes the potent anticholinesterase snake venom toxin, fasciculin (Fas; Karlsson et al., 1984) for more efficient and sensitive measurement of AChE densities (Anglister et al., 1998). Taken together, the quantitative EM-autoradiography studies revealed several-fold differences in synaptic AChE densities (~600–2500 sites per μm^2^ of postsynaptic surface) in muscles of different vertebrates (Rogers and Salpeter, 1969; Salpeter et al., 1972, 1978; Anglister et al., 1994b, 1998).

Although EM-autoradiography is a powerful tool for measuring densities, it does not offer the resolution required for fine localization of AChE. Thus, the precise distribution and location of the enzyme in the cleft could not be determined. Several basic questions remained to be resolved, such as whether and to what extent synaptic AChE is associated with pre- or postsynaptic surfaces within the cleft, or with the synaptic basal lamina (BL). It is well established that much of the synaptic AChE at the NMJ is associated with the BL (McMahan et al., 1978; Anglister et al., 1994a; for review see Massoulié and Millard, 2009), and is composed primarily of the asymmetric collagen-tailed AChE isoforms (A-AChE; for reviews see Legay, 2000; Massoulié, 2002; Rotundo, 2003; Massoulié and Millard, 2009). However, at least some of the synaptic AChE at the NMJs, and almost all the AChE at cholinergic synapses in the CNS, consists of a hydrophobic membrane-bound globular tetramer (G_4_-AChE; e.g., Fernandez et al., 1996; Perrier et al., 2002; Tsim et al., 2010), which is elevated by exercise (e.g., Gisiger et al., 1994; Blotnick and Anglister, 2016). Moreover, while part of the synaptic enzyme at the NMJ is provided by the muscle (Hall and Kelly, 1971; Vigny et al., 1976; Lømo et al., 1985; Anglister et al., 1994a), accumulated evidence indicates anterograde axonal transport of AChE (Di Giamberardino and Couraud, 1978), as well as a direct neuronal contribution (Anglister, 1991; Jiang et al., 2003; Mis et al., 2005; Tsim et al., 2010). The differences in AChE origin and isoform composition could result in differential localization within the synaptic cleft.

Another issue that demands fine resolution of AChE distribution is whether synaptic AChE is found only in the primary cleft (PC) or also in postjunctional folds (PJFs), and what is its precise distribution within the fold. This information is crucial for understanding the role of AChE in terminating synaptic transmission.

In the present study we developed new probes for high resolution EM-localization of AChE, based on conjugates of nanogold particles with the anticholinesterase polypeptide toxin, Fas. These probes were then used to determine the precise localization and distribution of AChE within the synaptic cleft of the intact vertebrate NMJ. We showed that AChE sites are located both in the PC and the PJFs, with most of the AChE being associated with the BL. AChE in the PC was found to be closer to the myofiber than to the nerve terminal, in keeping with its association with the BL. AChE in the PJF was distributed over its full length, with maximum density about half way down. This positioning of AChE should enhance efficient hydrolysis of acetylcholine (ACh), and prevent its rebinding to the nicotinic ACh receptors (nAChRs). The localization that we have established for normal vertebrate NMJs provides a benchmark for comparison in studies of NMJs following changes in physiological and pathological conditions.

## Materials and Methods

### Materials

Materials were purchased from Sigma-Aldrich Inc. (St. Louis, MO, USA), unless specified otherwise.

### Animals

Adult female C57BL mice (Jackson Laboratory, Bar Harbor, ME, USA) and *Rana pipiens* frogs (J. M. Hazen Frog Co., Alburgh, VT, USA) were housed, cared for, and treated under controlled conditions, using protocols approved by the ethics committee (IACUC) of the Hebrew University of Jerusalem. The Hebrew University is an AAALAC International Accredited Institute.

### Preparation of Probes

#### Radio-Labeled Fasciculin-2 (^125^I-Fas)

Fasciculin-2 (Fas) was obtained from Alomone Labs (Jerusalem, Israel). Fas (67 mg, 10 nmoles) was incubated (90 min) with 1 mCi [^125^I]-NaI (Amersham, Bucks, UK), 5 mg lactoperoxidase (Sigma, St. Louis, MO, USA) and 10 μl of 0.03% H_2_O_2_, in a total volume of 1 ml of 0.1 M phosphate buffer, pH 6.5, followed by isolation of the ^125^I-Fas product as described previously (Anglister et al., 1998). The ^125^I-Fas preparation was tested for its capacity to inhibit AChE activity by the colorimetric assay of Ellman et al. (1961), using acetylthiocholine as substrate, and samples of purified G_2_ AChE from *Torpedo californica* (Futerman et al., 1985), a gift from Dr. I. Silman (Weizmann Institute, Israel; detailed in Anglister et al., 1998).

#### Biotinylated Fasciculin (Fas-bio)

Fas was biotinylated in our lab using a NHS-kit (Molecular Probes, Eugene, OR, USA; Blotnick et al., 2012; Blotnick and Anglister, 2016).

#### Nanogold-Fas Conjugate

Nanogold was directly conjugated in our lab to Fas, using Sulfo-*N*-hydroxy-succinimido Nanogold (2025A; Nanoprobes, Yaphank, NY, USA); the conjugate was separated from the excess reagent by gel filtration on a Superdex-75 column (GE Healthcare Bio-Sciences, Pittsburgh, PA, USA).

#### Biotinylated α-Bungarotoxin (BTx-Biotin)

This conjugate (B-1196) was purchased from Molecular Probes (Eugene, OR, USA).

### Tissue Preparation

Adult female mice were anesthetized by intramuscular injection of chloral hydrate solution (50 mg/ml, 0.2 ml/25 g body weight), followed by an additional injection of 0.1 ml/25 g body weight 40 min later to ensure anesthesia throughout the procedure. The right sternomastoid muscle was exposed by an incision in the neck, and the skin was held back so as to create a well. Frogs, anesthetized by immersion in water containing 0.1% MS-222 (tricaine methane sulfonate; Sigma Chemical Co., St. Louis, MO, USA), and the cutaneous pectoris muscles were removed and pinned out in a Sylgard^®^-coated petri dish containing ice cold frog Ringer’s solution (50 ml, 116 mM NaCl, 20 mM KCl, 1.8 mM CaCl_2_, 10 mM sucrose, 1 mM Na-phosphate, pH 7.2; Anglister and McMahan, 1985).

### EM-Autoradiography

Labeling of mouse sternomastoid muscles followed our earlier protocol (Anglister et al., 1998). The experimental group included three mice. Briefly, the toxin solution–^125^I-Fas2, 150 μl of a 0.4 μM solution in mammalian Krebs**-**Ringer solution (118 mM NaCl, 4.7 mM KCl, 2.5 mM CaCl_2_, 1.2 mM MgSO_4_, 1.2 mM KH_2_PO_4_, 20 mM Hepes, 10 mM D-glucose, pH 7.4), containing 1 mg/ml BSA (Krebs-Ringer-BSA)—was applied topically to the well for 90 min, to ensure saturation of the muscles. After removal of the toxin solution the muscles were immediately rinsed with Krebs-Ringer-BSA. The mice were then sutured, and allowed to wake from the anesthesia, to provide for further wash-out of non-incorporated Fas during the following 2 h by the animal’s own blood flow. After the wash-out period the mice were re-anesthetized with an overdose injection of chloral hydrate, and perfused intracardially with 4% paraformaldehyde. The muscles were then excised, and fixed for a further 1 h in the paraformaldehyde, followed by washing with 0.067 M phosphate, pH 7.4, containing 5% sucrose (P-buffer). The endplate-rich region of each muscle was then excised, rinsed with P-buffer, post-fixed in 1% OsO_4_, block-stained with uranyl acetate, dehydrated in alcohol, and finally embedded in Polarbed 812 (Bio-Rad, Polaron, Cambridge, MA, USA), as described earlier (Fertuck and Salpeter, 1976; Anglister et al., 1998).

### EM-Nanogold

The procedure for labeling mouse sternomastoid AChE with nanogold probes was similar to that described above for EM-autoradiography, with the following differences: The toxin probe, Fas-biotin or Fas-nanogold, was applied topically to the exposed muscle in each of five mice (150 μl, 0.4 μM solution in Krebs-Ringer containing 1 mg/ml BSA) for 90 min. The toxin was removed, and the muscles were rinsed (5 × 3 min) with Krebs-Ringer-BSA. At this stage, the mice with muscles labeled by Fas-nanogold were sutured, and allowed to wake from the anesthesia, to provide for further wash-out of non-incorporated toxin-conjugate during the following 90 min by the animal’s own blood flow. The mice with muscles labeled by Fas-biotin were maintained under anesthesia, while the muscles were treated with 1.4 nm nanogold-streptavidin (Nanoprobes Inc., Yaphank, NY, USA) in Krebs-Ringer-BSA for 1 h at RT, and then rinsed (3 × 5 min) in Krebs-Ringer. The mice were then sutured and allowed to wake, to permit further wash-out of the muscles (see above). All mice were then re-anesthetized with an overdose injection of chloral hydrate, and perfused intracardially with 4% paraformaldehyde. Muscles were removed and re-fixed in the same fixative (1 h) followed by 1% glutaraldehyde and 4% paraformaldehyde for 30 min at RT. The muscles were then rinsed in P-buffer and soaked overnight at 4°C. The endplate-rich region of the muscle was excised and separated into small samples (1 mm × 2 mm). In all preparations, nanogold labeling was intensified with HQ-silver (Molecular Probes; 4 min in the dark), and stabilized with gold-toning (as specified in the Nanoprobes Instruction Manual). The specimens were then post fixed in 0.75% OsO_4_, block-stained with uranyl acetate, dehydrated in alcohol, embedded and thin-sectioned (100 nm) as described elsewhere (Anglister et al., 1994a,b, 1998; Blotnick and Anglister, 2016). In control experiments, exposed muscles were pretreated with unlabeled Fas (1 μM, 150 μl, 40 min), to saturate all binding sites on AChE. Subsequently the muscles were treated with the labeled-Fas sequence, fixed and processed as the experimental specimen. In additional control, exposed muscles were directly treated with nanogold-streptavidin (or just nanogold particles), omitting the Fas labeling altogether, and then continued as described above. No detectable gold-particles were observed at the NMJs of either of the control muscles preparations. The procedure for labeling frog cutaneous pectoris AChE (or AChR) and preparation of the specimens for EM, were basically as described above for mouse muscles with the following differences: Labeling was with higher concentration of Fas-nanogold (1 μM) because of a lower binding constant of Fas to frog AChE (Ki = 60 nM; Anglister, unpublished data) compared with Fas binding to mouse AChE (Ki = 15 pM; e.g., Anglister et al., 1998); all labeling, rinsing and fixation steps were done with isolated muscles (from three frogs) in a dish and with solutions isotonic with frog Ringer’s (as in Anglister et al., 1994a,b).

Electron microscopy and photography utilized a CM12 TEM 100 kV instrument (Phillips, Eindhoven, Netherlands). NMJs were identified and photographed at 23k× magnification.

### Data Acquisition and Statistical Analysis

**Figure 1:**
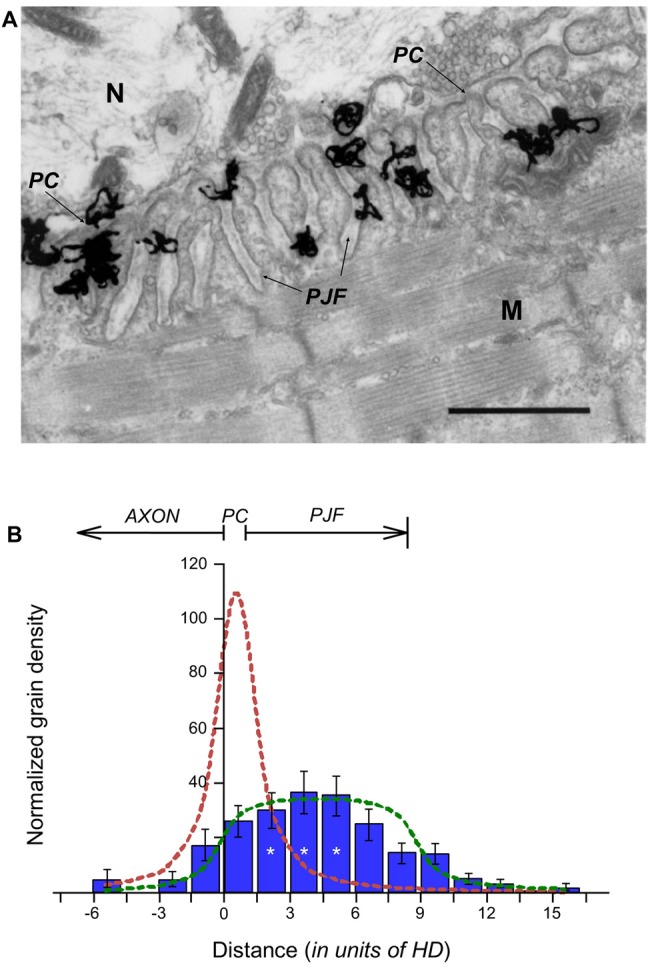
Distribution of Acetylcholinesterase (AChE) sites within the synaptic cleft. **(A)** EM-autoradiogram of mouse neuromuscular junction (NMJ) labeled with ^125^I-Fas. Mouse sternomastoid muscles were incubated with ^125^I-Fas (0.4 mM, 55 Ci/mmole) to inhibit AChE activity. They were then rinsed, fixed and processed for EM-autoradiography. Grains appeared almost exclusively at the NMJs. N, nerve; M, muscle. Arrows point to the primary cleft (PC) and to postsynaptic junctional folds (PJFs). Bar, 1 μm. **(B)** Radioactivity is distributed over the full depth of the PJFs. Grain density histogram (grains per unit area of autoradiogram) as a function of distance (in resolution units of HD = 80 nm; see, Salpeter et al., 1977) on either side of the axonal membrane (at 0), of 81 NMJs, three mice: left side into axon; right side into muscle. The bottom of the PJFs is on the average 11 HD (arrow), but with a large range; consequently, it is not as sharp a boundary as that provided by the axonal membrane. The reduced grain densities on both sides of the histogram are consistent with the radiation sources being within the PC and PJF, the variability in the depths of the PJFs and the actual spread of radiation under the experimental conditions (>3 HD). *, see text in “Results” section for detailed statistical parameter.

Computerized morphometry of the photographs for quantification of gold-particles, corresponding to the labeled AChE, and estimation of their distribution in the NMJs, utilized Adobe Photoshop CS4 software (Adobe Systems Incorporated, San Jose, CA, USA). The presynaptic axonal membrane and the opposing postsynaptic muscle membrane, including the PJFs, were outlined. The shortest distances of each gold particle from the axonal and muscle membranes were measured. In the same way, the locations of the BL boundaries adjacent to each gold particle were measured, taking the location of the presynaptic membrane as zero in the PC. Subsequently the distances measured in the PC were normalized by the width of the PC at the particle’s location. Those measured in the PJF were normalized by the width or length of the fold, as detailed in the specific experiments (Figures 3, 4). Measurements from electron micrographs of examined synaptic sites were collected for each mouse. Means were calculated by combining all synaptic sites per animal (81 NMJs, 3 mice in Figure 1; 28 NMJs, 5 mice in Figures 3, 4). One-way ANOVA, followed by the Tukey HSD posthoc method for multiple comparisons, was used to compare densities of labeled sites (whether nanogold particles or autoradiography grains) whether in the PC or the PJFs (Figures 1, 3, 4). Comparisons were also made using the student’s *t*-test with unequal variances (as indicated for specific experiments). Tests were available in Microsoft Office Excel Professional software (Microsoft Co., Redmond WA, USA), GraphPad Prism4 (GraphPad Software Inc., La Jolla CA, USA), and Statistica10 (StatSoft Inc., Tulsa OK, USA). The precise values of statistical parameters were detailed (*F, t, p*); the difference was considered significant when *p* < 0.05.

**Figure 2:**
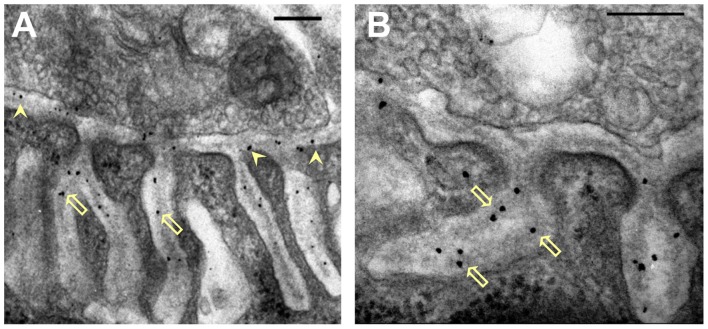
Distribution of nanogold-labeled AChE sites in the synaptic cleft of the mouse endplate. **(A,B)** Electron micrographs of cross sections through endplates of mouse sternomastoid muscles that had been incubated with Fas-biotin followed by 1.4 nm-nanogold-streptavidin and light silver intensification (details under “Materials and Methods” section). Gold particles (black) are seen over the PC (**A**, arrowheads) and PJFs, and are predominantly associated with the basal lamina (BL; **B**, hollow arrows). Bars, 0.2 μm.

**Figure 3:**
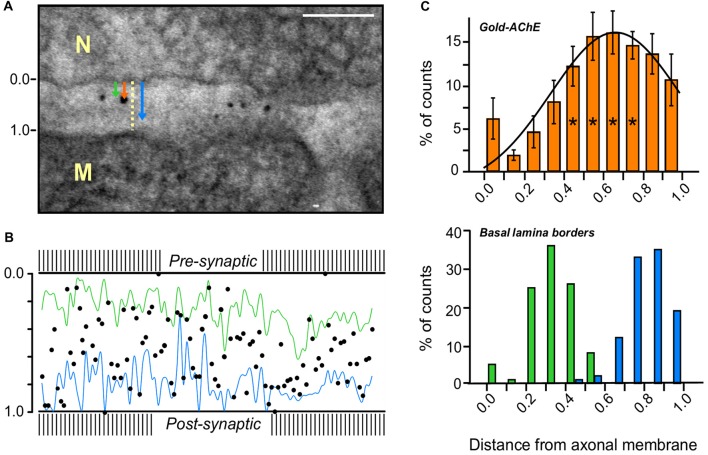
AChE distribution in the PC of a mouse sternomastoid endplate. **(A)** Paradigm for the measurement of the distribution of gold-labeled AChE sites in the PC. Distances from the axonal pre-synaptic membrane (0.0) of each gold particle (orange arrow), and of the boundaries of the BL on both sides of the particle (green and blue arrows), were measured and divided by the width of the PC at that site (yellow dashed line); Bar, 0.1 μm. **(B)** Cumulative display of the gold-labeled AChE sites (measured in **A**) in a normalized PC of mouse sternomastoid endplate (five mice, 28 NMJs). Most gold particles are found on the synaptic BL (blue and green lines connect the BL border measurements on either side) in the PC between the presynaptic axonal membrane (0.0) and the postsynaptic muscle membrane (1.0). **(C)** Quantitative distribution of the gold-labeled AChE sites across the width of the PC, from compiled data obtained as in **(A,B)**. Presynaptic axonal membrane, at *X* = 0.0, postsynaptic muscle membrane at *X* = 1.0. The histogram of the gold-particles (orange, upper panel) relative to the histograms of the BL boundaries (green-blue, lower panel) shows that the AChE sites are mainly spread over the BL in the PC. It should be noted that the AChE sites are predominantly closer to the postsynaptic muscle membrane than to the presynaptic nerve membrane. *, see text in “Results” section for detailed statistical parameters.

**Figure 4:**
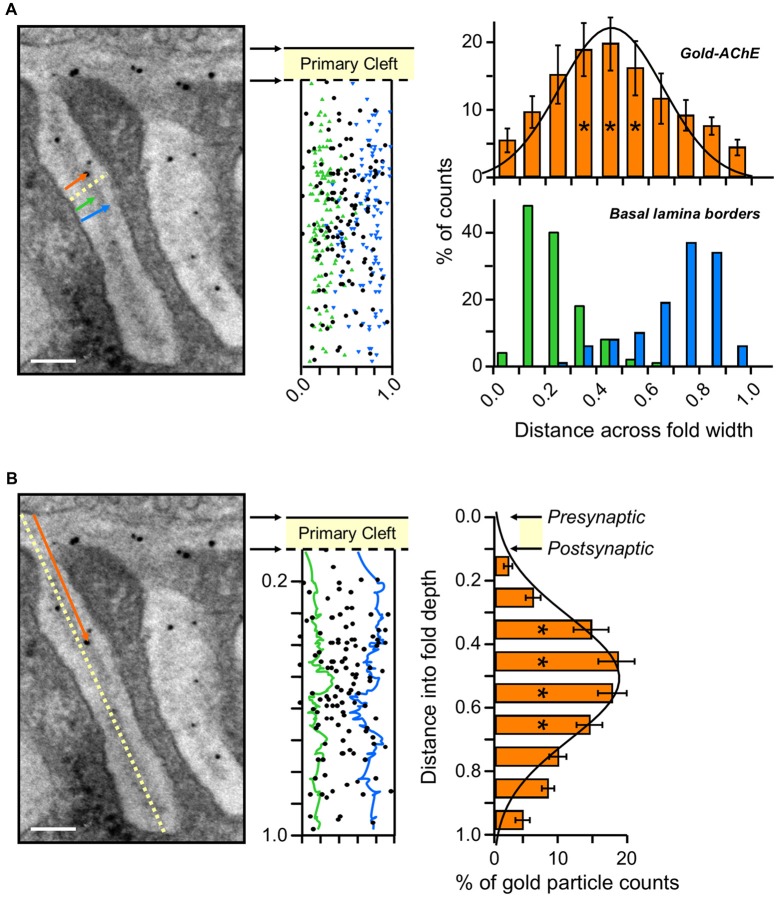
Gold-AChE distribution in the PJF. **(A)** Distribution across the width of the PJF. Left panel, measurement paradigm: distances from the myofiber fold membrane of each gold particle (orange arrow) and of the BL boundaries (green and blue arrows) were measured and divided by the width of the fold at that site (yellow dashed line). Center panel, compiled locations of the gold-labeled AChE sites (black) positioned in a normalized cleft within the adjacent measured BL boundaries (green and blue, as in **A**). Right panel, histograms of the compiled gold-labeled AChE sites (top, orange) relative to the adjacent BL boundaries (bottom, green and blue) reveal a spread of AChE sites over the BL within the PJF. Gaussian fit shows maximal concentration of sites at the center of the BL, with an even distribution towards either side. Fold width, *X* = 0.0 to *X* = 1.0. **(B)** Distribution along the long axis of the PJF. Left panel, measurement paradigm: The distance of each gold particle from the axonal membrane (orange arrow) was divided by the total depth of the fold (yellow dashed line). Center panel, compiled locations of gold-labeled AChE sites (black) in a normalized cleft within the outlined green and blue BL boundaries, marked above (**A**, center), and smoothed by moving average for convenient visualization. Right panel, histogram of the compiled data for the distances of the gold-labeled AChE sites from the axonal membrane (left panel) shows the distribution of AChE sites along the length of the PJF, with a maximum about halfway down. Data accumulated from micrographs of same NMJs as in Figure 3 (five mice, 28 NMJs). Axonal membrane, *X* = 0.0; Postsynaptic muscle membrane (top of fold), *X* = 0.1; Bottom of fold, *X* = 1.0. *, see text in “Results” section for detailed statistical parameters. Bars, 0.1 μm.

## Results

### Distribution of AChE Sites Within the Synaptic Cleft

In autoradiograms of mouse muscles in which AChE sites had been labeled with ^125^I-Fas, the grains that corresponded to the radio-labeled AChE were concentrated at the NMJs, and appeared over the PC and the PJFs, with grains being seen all the way into the folds (Figure 1A). To determine whether this was indeed due to AChE distribution throughout the PJFs, or whether AChE was located exclusively within the PC, and the grains over the folds were a consequence of radiation spread, the grain distribution in the autoradiograms was analyzed. All grains observed in the examined sternomastoid muscles (81 NMJs, three mice) were counted and tabulated against their distances from the axonal membrane. The distances were displayed in resolution units of “half distance” (HD), this being the distance from the radioactive source that encompasses 50% of the grains. HD is 80 nm for the isotope ^125^I used in our autoradiographic protocol (Salpeter et al., 1977). The histogram in Figure 1B shows a distribution that can arise only if the sources, i.e., the radiolabeled AChE active sites, are distributed over the junctional folds. Concentration of the sources solely within the PC would have produced a maximum grain density at 0–1 HD (0–80 nm), with a sharp and even decline on both sides (Figure 1B, dashed red theoretical line), as had been shown earlier for the distribution histograms of ^125^I-α-bungarotoxin-labeled AChRs (Fertuck and Salpeter, 1976). Thus, whereas AChR is concentrated at the crests of the folds, it is clear that a substantial percentage of the AChE sites are within the PJFs. The calculated uniform distribution of AChE within the fold appears to be similar, but not identical, to the experimental data (Figure 1B, compare the dashed green curve to the blue bars of the experimental histograms). Statistical analysis of the experimental data shows significant difference between the AChE site densities (one way ANOVA, *F*_(14,45)_ = 7.85, *p* = 0.000001). For simplification, the data were compared after pooling into three groups: −6 to −3 HD, 1.5–4.5 HD (center, * in Figure 1B) and 10.5–13.5 HD. One-way ANOVA (*F*_(2,24)_ = 184.26, *p* = 0.000000) followed by Tukey HSD test for multiple comparisons, show significant differences between the densities in the center group and each of the other edge groups (*p* = 0.000129). Still, due to the limitation imposed by radiation spread, autoradiographic examination is not suited for determination of the precise distribution, and a higher resolution technique was required.

### Nanogold Labeling of Acetylcholinesterase at Vertebrate Neuromuscular Junctions

**Figure 5:**
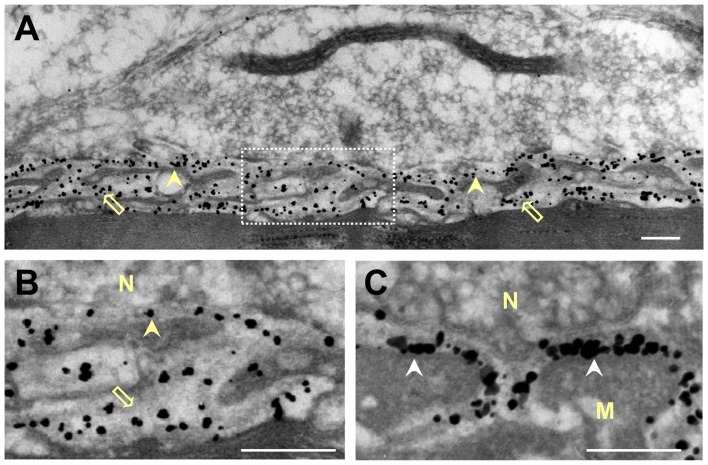
Distribution of gold-labeled AChE sites in the synaptic cleft of the frog NMJ. Frog cutaneous pectoris muscles were incubated with nanogold (1.4 nm) conjugated directly with Fas to label AChE **(A,B)**, or with biotin-α-bungarotoxin followed by nanogold-streptavidin to label nAChR **(C)**. **(A)** Nanogold-Fas conjugate labeled exclusively the NMJs, within both the PC and the PJFs. **(B)** An enlarged synaptic area (framed in **A**) shows the association of the gold-labeled AChE sites with the BL in both the PC and the PJFs. **(C)** In contrast, labeling of nAChR sites with the nanogold-α-bungarotoxin conjugate is seen to be at the crests and mouths of the PJFs. N, nerve; M, muscle; Arrowheads, labeled AChE in PC; Hollow arrows, labeled AChE in PJF; White arrowhead in **(C)**, labeled nAChR (on postsynaptic membrane). Bars, 0.2 μm.

To achieve higher resolution of AChE location at the synapse we prepared conjugates of Fas-nanogold or Fas-biotin (to be followed by streptavidin-nanogold, see “Materials and Methods” section) to label AChE at both mouse and frog NMJs. Nanogold-labeling was obtained selectively at the mouse NMJs (Figure 2), and practically nowhere else on the mouse muscle surface (examined at least 1 μm away from the NMJ; as in Anglister et al., 1998). Intense and selective nanogold-labeling was obtained also at frog NMJs (Figure 5), where AChE site-density is low relative to that in the NMJs of other vertebrates (Anglister et al., 1994b). Gold-labeled AChE sites were seen both in the PC and the PJFs of the mouse (Figure 2A, arrows) and frog NMJs (Figure 5A). Moreover, high-power examination indicated that the gold-particles are associated with the BL in the PC and the PJFs in both mouse and frog (Figures 2B, 5B, respectively).

### Localization of AChE in the PC

Nanogold-labeled AChE distribution in the PCs of mouse endplates was analyzed quantitatively to determine whether the AChE is located on the presynaptic membrane, the postsynaptic membrane, or the BL. For each nanogold particle in the PC, we measured the distances from the axonal membrane of the particle, and of the proximal and distal boundaries of the BL adjacent to it. These values were then normalized by the width of the cleft at that site (Figure 3A). The evaluated particles were positioned on a normalized PC (Figure 3B), and the data thus obtained were combined for subsequent analysis.

Quantitative analysis of the distribution across the width of the cleft (28 NMJs, five mice) presented in a histogram (Figure 3C) showed that >95% of the gold particles in the PC were on the BL (within the boundaries, Figure 3C, lower panel), rather than on the presynaptic or postsynaptic membranes, with maximal location closer to the myofiber face than to the axonal face, in keeping with the association with the BL (Figure 3C, upper panel). Statistical analysis (one-way ANOVA) indeed confirmed significant variation in particle distribution across the PC (*F*_(9,90)_ = 4.52, *p* = 0.000065). While the density in the region of the BL (columns at *x* = 0.5–0.8) was invariable (Tukey’s HSD test *p* = 0.998–1.000), it differed from the density at the edges. For simplicity, the data were compared after pooling into three groups across the PC: data at distances 0.1–0.3 (axonal-side), at 0.5–0.8 (center, * in Figure 3C) and at 0.9–1.0 (muscle-side). One-way ANOVA (*F*_(2,57)_ = 9.34, *p* = 0.00031) followed by Tukey HSD test for multiple comparisons, show significant increase in density in the center group compared with the axonal-side group (*p* = 0.00029) and a trend of increase compared with the muscle-side group (*p* = 0.087), indicating deviation in AChE sites distribution across the PC toward the muscle face. Indeed, further analysis of the distribution of the nanogold particles showed that significantly more particles are on the side of the BL midline facing the postsynaptic muscle membrane (62.3 ± 1.9%) than on the side facing the axonal membrane (37.7 ± 1.9%; *p* = 0.000000; two-tailed Student’s *t-Test*, *t* = 2.10).

### Distribution of AChE Within the PJFs

A similar procedure to that performed to determine the distribution of AChE sites in the PC was used to analyze the fine localization of AChE in the PJFs of the mouse endplate.

Figure 4 illustrates a PJF with nanogold-AChE labeling (black particles) and the definition of the co-ordinates. For each particle we measured its distance from the muscle membrane (within the fold, orange arrow in Figure 4A, left), and its distance from the axonal membrane (orange arrow in Figure 4B, left). We further measured the position of the BL edges within the fold (blue and green arrows, Figure 4A, left). Finally, we measured the width of the fold (yellow dashed line, Figure 4A, left) and its length (yellow dashed line, Figure 4B, left). To merge all the data points we normalized all the width measurements by the fold width, and the particle depths by the fold length. The normalized data (from 23 NMJs, five mice) were summarized and displayed on a model that maintains the ratios between the mean width and length of the fold and the width of the PC (Figures 4A,B, center). Most of the gold particles (92.6% ± 0.6) are within the boundaries of the BL, between the margins, and are found along the whole length of the fold.

Quantitation of AChE across the width of the PJF is shown in a histogram of the gold particles set above a histogram of the BL margins (Figure 4A, right). AChE is spread over the BL. Gaussian fit illustrates central concentration of the particles within the BL across the fold. Statistical analysis confirms significant variation in particle distribution across the PJF (one-way ANOVA, *F*_(9,70)_ = 5.48, *p* = 0.000011). The data were further compared after pooling into three groups: densities at 0–0.3, 0.3–0.6 (center, * in Figure 4A, top right) and 0.7–1.0. One-way ANOVA (*F*_(2,69)_ = 24.65, *p* = 0.000000) followed by Tukey’s HSD test for multiple comparisons show significantly higher density in the center group, over the BL, compared with the particle density on either 0–0.3 (*p* = 0.000140) or 0.7–1.0 (*p* = 0.000112) sides.

Analysis of the particle distribution along the fold (Figure 4B, right) shows that AChE is located along the whole length of the fold, with a maximal density about half way down, as illustrated by the Gaussian fit of the data (Figure 4B, right). Statistical analysis strengthens this observation (one-way ANOVA, *F*_(9,70)_ = 5.48, *p* = 0.000011). Further comparison was done after pooling the data into three groups, at distinct distances from the axonal membrane into the fold: 0–0.3 (top of the fold), 0.3–0.7 (mid-fold, * in Figure 4B, right) and 0.7–1.0 (bottom of the fold). One-way ANOVA (*F*_(2,77)_ = 39.33, *p* = 0.000000), followed by Tukey’s HSD test for multiple comparisons, confirmed significant elevation of particle density at mid-fold, compared with densities at the top or bottom of the fold (*p* = 0.000109).

Because the frog NMJ has a distinct shape, and displays relatively low AChE density compared with the mammalian endplate, but similar nAChR density (Anglister et al., 1994b), it was important to establish AChE location relative to that of nAChR in the frog synaptic cleft. Frog cutaneous pectoris muscles were incubated with nanogold conjugated directly to Fas to label AChE, or with biotinylated-α-bungarotoxin (followed by nanogold-streptavidin) to label nAChR. The directly labeled gold-Fas probe was used in order to obtain conspicuous labeling, which is less suitable for quantitative assessments due to particle overlap, but can clearly distinguish between the locations of AChE and nAChR. Figure 5 demonstrates the distinctive shapes of frog and mouse PJFs (compare Figures 5A and 2A, respectively). While the labeled nAChR sites appear at the crests and mouth of the folds (Figure 5C, white arrowheads), the labeled AChE sites appear on the BL in both the PC and the PJFs of the frog NMJ (marked by yellow arrowheads and hollow arrows in Figures 5A,B, respectively). The flattened shape of the frog PJFs makes it problematic to assess quantitatively the distribution of AChE within them. However, there appears to be a markedly high density of AChE sites in the flattened expanded bottom of the folds (Figure 5B, hollow arrow).

## Discussion

The fine localization and distribution of AChE in the synaptic clefts of vertebrate NMJs was resolved in this study by employing a novel nanogold-EM method. We showed that: (1) AChE sites are located both in the PC and the PJFs; (2) Most of the AChE in both the PC and PJF is on the BL; (3) AChE in the PC is on average positioned closer to the myofiber than to the nerve terminal surface; and (4) AChE sites are distributed over the full length of the PJF, with maximum density at its midpoint. The labeling of AChE with 1.4 nm-nanogold particles in the PC and PJFs utilized the anti-AChE toxin Fas, which binds specifically and tightly to AChE (Anglister et al., 1998; Martinez-Pena y Valenzuela et al., 2005) and is linked to nanogold particles (directly or via a biotin-streptavidin complex). The resolution of the nanogold labeling is greatly superior to that achieved by the previously employed EM-autoradiography technique (Anglister et al., 1998)—~2 nm for the nanogold-probe, compared to >100 nm for autoradiography, due to the spread of grains from the radio-iodinated bound probe (HD = 80 nm, half distance of grains from source, Figure 1). Thus, nanogold-Fas labeling could unequivocally demonstrate that the labeled AChE was associated with the BL. Furthermore, nanogold labeling of the tissue and subsequent processing and analysis were much easier, safer and less time consuming than using EM-autoradiography.

### AChE in the PC

As already mentioned, nanogold-labeled AChE was found primarily, if not exclusively, on the BL of both the PC and PJFs. In the PC, the enzyme was distributed closer to the postsynaptic (muscle) surface of the BL than to the presynaptic (neuronal) surface, and clearly not adjacent to the nerve terminal membrane as reported by Bernard et al. (2011). Possibly, the much larger immunoprobes (primary and secondary conjugated antibodies) used in that report did not penetrate the BL structures as well as the much smaller toxin probe that we used. In addition, the different locations observed may reflect distinct types of AChE isoforms (e.g., A-form vs. globular hydrophobic G4). It is well established that AChE at NMJs can be produced by both muscle (Hall and Kelly, 1971; Vigny et al., 1976; Anglister and McMahan, 1985; Lømo et al., 1985; Rotundo et al., 2005) and nerve (Anglister, 1991; Jiang et al., 2003; Mis et al., 2005; Tsim et al., 2010). Synaptic AChE is composed primarily of the large asymmetric collagen-tailed isoforms (mainly A_12_-AChE; reviewed in Legay, 2000; Massoulié, 2002; Rotundo, 2003; Massoulié and Millard, 2009). It is unlikely that such large molecules can travel a large distance from their site of secretion into the cleft, which is packed with complex structures of the BL, before their association with it. In fact, extracellular A-AChE in muscle cultures was shown to be concentrated over the nuclei of origin (Rossi and Rotundo, 1992). Our observation that the AChE in the PC is located closer to the muscle, and that it is also found along the length of the PJFs, is consistent with substantial production of synaptic AChE by muscle. After subunit synthesis and assembly in the rough endoplasmic reticulum and Golgi apparatus, it is secreted to its functional location at the synapse to be inserted in “parking” sites on the synaptic BL in association with heparan sulfate, perlecan and MuSK (Rotundo et al., 1997; Peng et al., 1999; Cartaud et al., 2004; for reviews see Rotundo et al., 2005; Massoulié and Millard, 2009). This efficient insertion is enhanced by activity and by intracellular Ca^2+^ (Martinez-Pena y Valenzuela et al., 2005). However, it cannot be ruled out, although not yet demonstrated, that there may be active transport of secreted A-AChE within the BL to selective target sites.

Nevertheless, as already mentioned, some of the AChE in the PC may originate from the nerve (Di Giamberardino and Couraud, 1978; Anglister, 1991; Jiang et al., 2003; Mis et al., 2005; Tsim et al., 2010). In this context it should be noted that a small fraction (5.9 ± 2.2%) of the total AChE in the PC was detected adjacent to the neuronal membrane (Figure 3C). This fraction may reflect nerve-derived and possibly membrane-bound G_4_-AChE attached to the presynaptic membrane (Bernard et al., 2011).

AChE at the intact adult mammalian NMJs is very stable, with t_1/2_ values for degradation of ~20 days (Kasprzak and Salpeter, 1985) or 12 days (Krejci et al., 2006), reported using different techniques. The issue of the regulatory mechanisms involved in maintenance of AChE at the NMJ at the required densities and loci has yet to be resolved. In this context, evidence has been provided that AChE is removed by internalization in the same pool as the postsynaptic membrane-bound nAChR (Krejci et al., 2006); this is consistent with the proximity to the postsynaptic muscle membrane that we observe. However, metabolic processes within the extracellular matrix may also be involved (Sprangers and Everts, 2017).

### AChE in the PJFs

Already many years ago histochemical staining for AChE at NMJs revealed reaction products in the PJF as well as in the PC (Koelle and Friedenwald, 1949; Couteaux, 1955, 1998). However, it was not certain whether the stain reflected the true location of the enzyme in the PJF or was a consequence of migration of the reaction product prior to precipitation. Here we first showed that EM-autoradiography of mouse muscles labeled for AChE with ^125^I-Fas also showed the presence of grains over the PJF (Figure 1A), with increased density halfway down the fold. The grain distribution observed could not be ascribed to AChE within the PC or at the crests of the folds (Figure 1B, simulated red plot). Yet, as already pointed out, EM-autoradiography lacks the resolution required to distinguish between homogeneous distribution of AChE along the entire length of the fold (Figure 1B, simulated green plot) or a selective accumulation of AChE halfway down the fold, as seen in the experimental histogram (Figure 1B). This issue was resolved by using the nanogold-EM technique. The higher resolution that it achieved showed that: (1) AChE is definitely seen along the entire length of the PJF (Figure 2); (2) It is found within the BL (Figure 4A); and (3) There is indeed a maximal density of AChE halfway down the fold (Figure 4B).

The distribution of AChE in both the PC and the PJF raises several questions: First of all, does the location reflect consequences of the metabolism of AChE, its insertion and removal under conditions of normal maintenance? In the previous section we addressed the issue of cellular AChE location in the PC with respect to its cellular origin, muscle or nerve, as well as its removal during physiological maintenance. As already mentioned, it is not known how AChE is inserted and removed from the PJF, and, more specifically, how its uneven distribution is generated and maintained. Whereas the nAChRs accumulate at the crests of the PJFs (Fertuck and Salpeter, 1974, 1976), and sodium channels at their bottoms (Flucher and Daniels, 1989), the high density of AChE mid-fold may be due to preferable insertion at this location. Alternatively, there may be uniform insertion throughout the fold but accelerated removal of AChE at the extremities. The options of insertion either at the top or at the bottom, followed by migration along the fold, appear implausible in view of the observed distribution.

Do the distributions observed have a physiological rationale? The location in the PC, closer to the postsynaptic surface, may ensure more efficient hydrolysis of ACh bouncing off the nAChRs, thus reducing rebinding and desensitization that might otherwise compromise muscle response (Katz and Miledi, 1973). Similarly, a high concentration of AChE halfway down the mouse PJF, and near the bottom of the flattened frog PJF, may serve as a safety barrier to prevent ACh from accumulating in the fold, and diffusing back to activate nAChRs between signals.

## Conclusion

The optimal function of a given cholinergic synapse demands a precise localization and density of AChE within the synaptic cleft. Conjugates of Fas can be utilized to assess the location and density of AChE in cholinergic synapses, as was shown here for vertebrate NMJs. While the densities of AChE at NMJs differ between mouse and frog fast twitch muscles, the localization is similar despite their rather different geometries. AChE molecules are located both in the PC and the PJFs, with most of the enzyme being associated with the BL at both locations. In the PC the bulk of the AChE is closer to the surface of the muscle than to the presynaptic membrane, in keeping with its association with the BL. In the PJFs, AChE is distributed over the full length of the folds, with a maximum density halfway down the fold. The distributions and densities of the AChE molecules serve to optimize hydrolysis of ACh molecules dissociating from nAChRs, thus eliminating deleterious accumulation and rebinding. The successful application of the Fas conjugates developed for the study of normal intact NMJ, can be extended for the study of other synapses in both the PNS and the CNS under physiological or pathological conditions.

## Author Contributions

LA designed the project and the experiments and obtained funding. Both EB-R and LA conducted the experiments, acquired and analyzed the data and prepared and approved the manuscript.

## Conflict of Interest Statement

The authors declare that the research was conducted in the absence of any commercial or financial relationships that could be construed as a potential conflict of interest.

## Abbreviations

ACh: acetylcholine
AChE: acetylcholinesterase
BL: basal lamina
Fas: fasciculin 2
nAChR: nicotinic acetylcholine receptor
NMJ: neuromuscular junction
PC: primary cleft
PJF: postjunctional fold
PJM: postjunctional membrane.
